# Prostate Involvement in a Patient with Follicular Lymphoma

**DOI:** 10.4274/tjh.2017.0181

**Published:** 2017-12-01

**Authors:** Seda Yılmaz, Sinan Demircioğlu, Özlen Bektaş, Özcan Çeneli, Sıdıka Fındık

**Affiliations:** 1 Necmettin Erbakan University Meram Medicine Faculty, Department of Hematology, Konya, Turkey; 2 Necmettin Erbakan University Meram Medicine Faculty, Department of Pathology, Konya, Turkey

**Keywords:** Follicular lymphoma, Extranodal, Prostatic involvement

## To The Editor,

While extranodal involvement is observed in 50% of cases of non-Hodgkin’s lymphoma, prostatic involvement is rare. Prostatic lymphoma accounts for 0.09% of all prostate neoplasms and 0.1% of all non-Hodgkin’s lymphomas [1].

**Figure 1:**
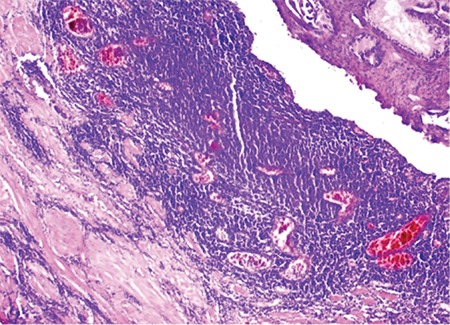
Diffuse lymphocytic infiltration.

Our patient was monitored for 4 years and had stage 4BS follicular lymphoma (bone marrow involvement; mesenteric lymph nodes in the abdomen, the largest of which was measured as 9x4 cm; cervical and mediastinal lymph nodes; and splenomegaly and B symptoms) at the time of diagnosis. He received CVP (cyclophosphamide, vincristine, prednisolone), CHOP (cyclophosphamide, adriamycin, vincristine, prednisolone), and gemcitabine therapy, respectively, and had lower urinary tract symptoms during follow-up. A hypertrophic prostate was palpated during the physical examination. The prostate-specific antigen (PSA) level was measured to be 8.3 (normal range: 0-4) ng/mL. Urinary analysis showed microscopic hematuria. Ultrasound examination detected a prostate volume of 60 mL. Transurethral resection of the prostate (TUR-P) pathology results showed a diffuse lymphocytic infiltration and positive staining for CD20, CD10, CD5, and BCL-2 (Figure 1). The symptoms of the patient regressed after treatment with rituximab plus bendamustine.

Prostate cancer is the most common cancer among men worldwide. There were 1,618,000 cases with 366,000 deaths in 2015 [2]. Prostatic lymphoma is a rare condition that accounts for 0.09% of all prostate neoplasms. While extranodal involvement is observed in about 50% of cases of non-Hodgkin’s lymphoma, prostatic involvement is rare. The usual clinical manifestations of prostatic involvement in lymphomas are lower urinary tract symptoms and acute urinary retention. High serum PSA levels are not typical for prostatic lymphoma. Our patient presented with high PSA levels.

A study that investigated prostate materials from 4831 subjects determined lymphoma in 29 subjects (0.6%). Eleven (0.23%) subjects had a history of concurrent lymphoma [3]. In patients with prostate cancer, the incidence of non-Hodgkin’s lymphoma of the prostate was observed to be 0.2% in a series of 4319 radical prostatectomy cases [4] and 1.19% in another series of 1092 cases [5].

In conclusion, prostatic lymphoma is clinically difficult to distinguish from benign prostatic hyperplasia and prostatic carcinoma as it occurs in the same age group and presents with similar symptoms; thus, the histopathological and immunohistochemical findings in TUR-P material are important. Early and appropriate treatment improves the patient’s quality and length of life.
